# A Hybrid Domain Image Encryption Algorithm Based on Improved Henon Map

**DOI:** 10.3390/e24020287

**Published:** 2022-02-17

**Authors:** Yong Chen, Shucui Xie, Jianzhong Zhang

**Affiliations:** 1 School of Communication and Information Engineering, Xi’an University of Posts and Telecommunications, Xi’an 710121, China; 13253700106@163.com; 2 School of Science, Xi’an University of Posts and Telecommunications, Xi’an 710121, China; 3 School of Mathematics and Statistics, Shaanxi Normal University, Xi’an 710119, China; jzzhang@snnu.edu.cn

**Keywords:** image encryption, improved Henon map, integer wavelet transform, double sandwich structure, SHA-512

## Abstract

A hybrid domain image encryption algorithm is developed by integrating with improved Henon map, integer wavelet transform (IWT), bit-plane decomposition, and deoxyribonucleic acid (DNA) sequence operations. First, we improve the classical two-dimensional Henon map. The improved Henon map is called 2D-ICHM, and its chaotic performance is analyzed. Compared with some existing chaotic maps, 2D-ICHM has larger parameter space, continuous chaotic range, and more complex dynamic behavior. Second, an image encryption structure based on diffusion–scrambling–diffusion and spatial domain–frequency domain–spatial domain is proposed, which we call the double sandwich structure. In the encryption process, the diffusion and scrambling operations are performed in the spatial and frequency domains, respectively. In addition, initial values and system parameters of the 2D-ICHM are obtained by the secure hash algorithm-512 (SHA-512) hash value of the plain image and the given parameters. Consequently, the proposed algorithm is highly sensitive to plain images. Finally, simulation experiments and security analysis show that the proposed algorithm has a high level of security and strong robustness to various cryptanalytic attacks.

## 1. Introduction

With the development of the information revolution, network technology has been rapidly popularized. As one of the critical carriers of information exchange in network technology, digital image plays an important role in our daily life, and its transmission security problem has been widely concerned. Therefore, digital image encryption arises at the historic moment. Image encryption can be used in application scenarios based on computer vision, such as medical vision [1,2,3], secure surveillance framework for Internet of Things [4], and biometrics [5]. While the digital image has the characteristics of large amount of data, high redundancy, and strong correlation between pixels [6], the encryption algorithms designed for text information, such as the Data Encryption Standard (DES) and Advanced Encryption Standard (AES), are unsuitable for image encryption scenarios [1,7].

In recent years, with the in-depth study of chaos theory, the unique properties of chaotic maps have been explored, such as pseudorandomness, ergodicity, nonperiodicity, and high sensitivity to initial values. These properties make the chaos-based image encryption algorithms can exhibit a good ability to protect image data. So far, the image encryption algorithms based on chaotic systems have been widely studied [8,9,10,11,12,13,14,15,16]. In [8], an image encryption algorithm based on random integer cycle shift and logistic map is proposed. Zhao et al. [9] proposed a dynamic block image encryption algorithm based on variable-length secret key and modified Henon map. Zhao et al. [10] proposed a chaotic encryption algorithm based on long short-term memory artificial neural networks (LSTM-ANN). In the proposed scheme, the chaotic sequence used in the encryption algorithm is constructed by the LSTM-ANN deep learning network. Chai et al. [11] proposed a chaotic encryption algorithm based on generative adversarial network (GAN), convolutional neural network (CNN), and denoising network. In the proposed algorithm, the deep learning reconstruction scheme based on GAN improves the robustness of the encryption algorith, and the CNN denoiser improves the visual expression of the decrypted image. In [12], a color image compression–encryption scheme based on autoencoder is proposed, where the encrypted image is losslessly compressed by unsupervised autoencoder deep learning networks and this can speed up the transmission. In [13], a chaotic encryption scheme based on genetic algorithm is proposed. Due to the inherent advantages such as high parallelism, huge information density, and ultralow energy consumption, deoxyribonucleic acid (DNA) computing attracts the attention of cryptographers. Therefore, various algorithms combining chaotic systems and DNA computing have been proposed. For instance, Wang et al. [17] proposed an image encryption algorithm based on a six-dimensional hyperchaotic system and DNA encoding. El-Shafai et al. [2] proposed a medical image encryption algorithm using the DNA–chaos cryptosystem. Suri et al. [18] proposed an image encryption approach based on coupled map lattice, DNA, and multiobjective genetic algorithm. Furthermore, with increasing demand for high-quality images, image compression techniques have become an effective way to save memory space and transmission bandwidth. As a result, some scholars have introduced image compression techniques to encryption systems, such as compressed sensing [6,19,20], self-encoder [12,21,22], cosine transform [20,23], and wavelet transform [3,24,25,26,27], etc.

In [2,8,9,13,14,15,16,17,18], several encryption schemes based on spatial domain are proposed. Image spatial domain encryption is fidelity encryption. In some spatial image encryption algorithms, the overly simple scrambling–diffusion schemes cannot effectively break the strong correlation of the plain image, making the algorithms vulnerable to chosen-plaintext attacks. Therefore, some researchers have designed multiround encryption structures to enhance the security level, which leads to inefficient encryption. However, for frequency domain encryption schemes, each change of coefficients in the transform domain leads to the change of all pixel values in the image spatial domain, and some scholars have shifted research directions to the more efficient frequency domain. Belazi et al. [24] proposed a novel image encryption scheme based on chaotic system and lifting wavelet transform. In [28], a new method of image encryption using fractional Fourier transform is proposed. With the emergence of encryption algorithms based on the spatial and frequency domains, hybrid domain encryption algorithms are proposed. Hybrid domain encryption, which combines the fidelity of spatial domain algorithms and the efficiency of frequency domain algorithms, provides high-level security. Aashiq et al. [3] proposed a medical image encryption method based on a chaos–DNA–IWT (integer wavelet transform) combined approach. However, the diffusion algorithm in this paper did not consider to employ bit-level diffusion, which has better diffusion performance. Luo et al. [25] proposed an encryption scheme using the IWT. In this paper, the authors used spatiotemporal chaos to diffuse low-frequency subbands and kept the high-frequency subbands unchanged. The diffusion algorithm did not take the full information of the image into account.

Based on the above analysis and to move beyond, we proposed a hybrid domain image encryption algorithm based on improved Henon map. The main contributions of this paper are summarized as follows:

(1) We improve the classical two-dimensional (2D) Henon map. The improved Henon map is briefly called 2D-ICHM. The analyses of dynamical properties show that 2D-ICHM has more complex chaotic behavior and is more suitable for image encryption scenarios.
(2) The proposed algorithm adopts a double sandwich structure based on diffusion–scrambling–diffusion and spatial domain–frequency domain–spatial domain. Specifically, the diffusion and scrambling operations are performed in the spatial and frequency domains, respectively, which provides a high level of security.
(3) To enhance plaintext sensitivity, the system parameters and initial values of chaotic mapping are obtained by the secure hash algorithm-512 (SHA-512) hash value of the plain image and the given parameters. Therefore, the proposed algorithm is highly related to plain image.

The remainder of the paper is organized as follows. In Section 2, we introduce the research status of the chaotic system. In Section 3, the 2D-ICHM is proposed and the dynamic performance is analyzed. In Section 4, related knowledge is introduced. In Section 5, we describe the proposed image encryption algorithm in detail. In Section 6, the simulation results are given. In Section 7, security analyses are presented. Finally, the conclusion of this paper is reported in Section 8.

## 2. Chaotic System

Chaotic systems are often used to design image encryption algorithms, due to their numerous excellent intrinsic characteristics, including unpredictability, aperiodicity, and pseudorandom behaviors [29,30]. In the image encryption algorithm, chaotic sequences generated by chaotic systems are often used in the process of image scrambling and diffusion. Chaotic systems are categorized as one-dimensional (1D) and high-dimensional (HD) systems, which have been a hot research topic for scholars. The classical 1D chaotic systems have the logistic, sine, and tent maps [31]. Due to the low complexity and easy predictability of 1D chaotic maps, the chaotic sequences generated by such maps are less stochastic and cause a number of security risks in image encryption processing. The HD chaotic systems have larger parameter space and more complex structure than the 1D chaotic systems, making the behavior of chaotic sequences more difficult to predict and more suitable for image encryption theoretically. However, chaotic systems with too high dimensions are not suitable for designing real-time image encryption systems, as they lead to intensive calculations and high implementation costs. The 2D chaotic systems, with higher complexity and lower implementation cost, provide a balance of chaotic performance and implementation cost. Hence, our scheme chooses to use 2D chaotic systems.

**Figure 1:**
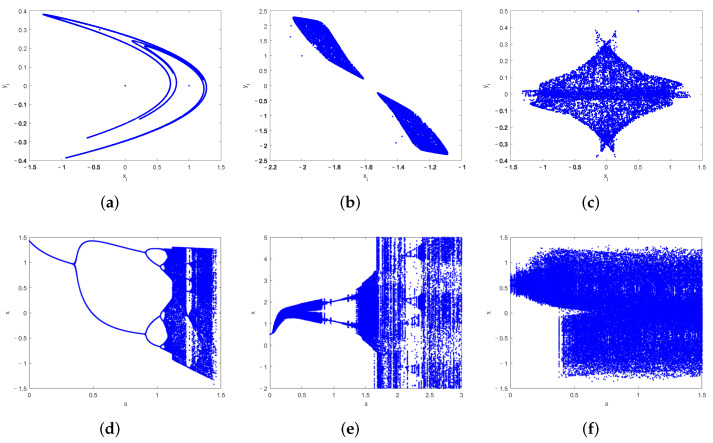
The phase portraits and bifurcation diagrams. Phase portraits: (**a**) 2D-CHM with $\left(a,b\right)=\left(1.4,0.3\right)$, (**b**) 2D-SM with $\left(a,b\right)=\left(0.7,3.8\right)$, (**c**) IHM with $\left(a,b\right)=\left(1,0.3\right)$, r=0.1; bifurcation diagrams: (**d**) the 2D-CHM with b=0.3, (**e**) 2D-SM with b=3.8, (f) IHM with b=0.3, r=0.1.

The classical 2D chaotic systems include cat map, standard map, Henon map, etc. [32,33]. In recent years, some weak chaotic characteristics of the classical 2D chaotic systems have been pointed out, such as small parameter space, discontinuous chaotic intervals, and poor pseudorandomness. Thus, researchers have made some improvements or proposed new 2D chaotic systems [34,35,36,37]. Hua et al. [34] proposed a new two-dimensional sine logistic modulation map based on a logistic map and a sine map. Zhu et al. [35] constructed a new two-dimensional chaotic system by using logistic and sine maps. Bao et al. [36] proposed a novel two-dimensional sine map (2D-SM) with a simple algebraic structure. A color image encryption algorithm using the improved Henon map (IHM) was proposed by Gao [37]. Figure 1 shows the phase portraits and bifurcation diagrams of the classical 2D Henon map (2D-CHM), 2D-SM, and IHM. The phase portrait and bifurcation diagram are the most common indicators to identify chaotic states. The phase portrait can represent the reciprocating nonperiodic motion characteristics of chaotic systems. The bifurcation diagram can clearly reflect the period-doubling bifurcation phenomenon and parameter range of chaotic systems, etc. As shown in Figure 1a–c, the motion trajectories of 2D-CHM, 2D-SM, and IHM are not uniformly distributed, indicating they have weaker randomness. As shown in Figure 1d–f, the 2D-CHM, 2D-SM, and IHM have discontinuous chaotic intervals and a small range of parameters. Therefore, it is crucial to design a 2D chaotic system with better chaotic performance.

## 3. Improvement of the Classical Two-Dimensional Henon Map

In this section, we give the definition of the 2D-ICHM and analyze its dynamical behavior. Further, comparison of the dynamical behavior of 2D-ICHM, 2D-CHM, 2D-SM, and IHM is considered.

### 3.1. Definition of 2D-ICHM

Henon map [38], a simple 2D discrete chaotic system, was introduced by Henon in 1976, which is defined as

(1) $$\left\{\begin{array}{c} x\left(n+1\right)=1+y\left(n\right)-ax{\left(n\right)}^{2},\\ y\left(n+1\right)=bx\left(n\right), \end{array}\right.$$

where $\left(x\left(n\right),y\left(n\right)\right)\in R^{2}$ are the state values of system, a∈[0,1.4], and b∈[0,0.3] are control parameters.

When a=1.4 and b=0.3, the 2D-CHM has the maximum Lyapunov exponent (LE), showing a most significant chaotic behavior. However, the 2D-CHM has some disadvantages, such as simple chaotic behavior and discontinuous chaotic intervals. In order to overcome the above shortcomings, we improve the 2D-CHM to 2D-ICHM, defined as follows:

(2) $$\left\{\begin{array}{c} x\left(n+1\right)=\cos\left(1-ax{\left(n\right)}^{2}\right)+e^{by{\left(n\right)}^{2}},\\ y\left(n+1\right)=\sin\left(x{\left(n\right)}^{2}\right), \end{array}\right.$$

where *a* and *b* are control parameters.

### 3.2. Chaotic Performance of 2D-ICHM

In order to verify the chaotic performance of the 2D-ICHM, the following analyses are discussed in terms of phase portrait, bifurcation diagram, Lyapunov exponent, approximate entropy, NIST SP800-22 test, and 0–1 test.

(1) Phase portrait and bifurcation diagram

**Figure 2:**
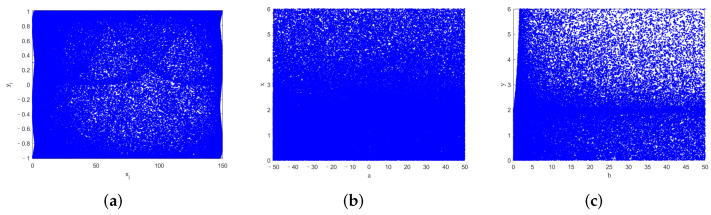
The phase portrait and bifurcation diagrams of the 2D-ICHM. (**a**) Phase portrait, (**b**) bifurcation diagram of output *x*, and (**c**) bifurcation diagram of output *y*.

Figure 2a is the phase portrait of 2D-ICHM with initial values $\left(x\left(0\right),y\left(0\right)\right)=\left(0.3,0.3\right)$, and the control parameters $\left(a,b\right)=\left(5,5\right)$. Figure 2b,c are the bifurcation diagrams of 2D-ICHM with $a\in \left(-50,50\right)$, b=5, and with a=5, $b\in \left(0,50\right)$, respectively.

As observed in Figure 1a–c and Figure 2a, the attractor structures of the 2D-CHM, 2D-SM, IHM, and 2D-ICHM are different. The attractor of 2D-ICHM is a noiselike pattern. It indicates that 2D-ICHM has better ergodicity. As can be seen from Figure 1d–f and Figure 2b,c, the bifurcation diagrams of the *x* and *y* components of the 2D-ICHM are also noiselike patterns, where the control parameters $a\in \left(-50,50\right)$ and $b\in \left(0,50\right)$. It indicates that 2D-ICHM has a larger parameter space and continuous chaotic range. Taken together, 2D-ICHM has a more complex chaotic behavior and is suitable for image encryption systems.

(2) Lyapunov exponent

The LE can be used to evaluate the chaotic behavior of dynamical systems. It can reflect the average exponential rate of separation or aggregation between adjacent trajectories [39]. The number of LEs is equal to the dimension of the chaotic system, which means that 2D chaotic systems have two LEs. A map has chaotic behavior when there is one positive LE value. The chaotic behavior of the map becomes more complicated as its LE increases. LE can be calculated using the Qatari Rial (QR) decomposition algorithm [40], which is defined as follows.

(3) $$\begin{array}{cc} & =[J_{M}J_{M-1}\cdots J_{2}\left(J_{1}Q_{0}\right)]\\ \; & =qr\left[J_{M}J_{M-1}\cdots J_{3}\left(J_{2}Q_{1}\right)\right]\left[R_{1}\right]\\ \; & =qr\left[J_{M}J_{M-1}\cdots J_{i}\left(J_{i-1}Q_{i-2}\right)\right]\left[R_{i-1}\cdots R_{1}\right]\\ \; & =\cdots \\ \; & =Q_{M}\left[R_{M}\cdots R_{2}R_{1}\right]\\ \; & =Q_{M}R, \end{array}$$

where qr[·] is the QR decomposition function, *J* is the Jacobian matrix of the chaotic map, and *M* is the number of iteration. Then, LE is calculated by

(4) $$LE_{v}=\frac{1}{M}\sum_{i=1}^{M}\ln\left|R_{i}\left(v,v\right)\right|,$$

where v=1,2,⋯,n.

**Figure 3:**
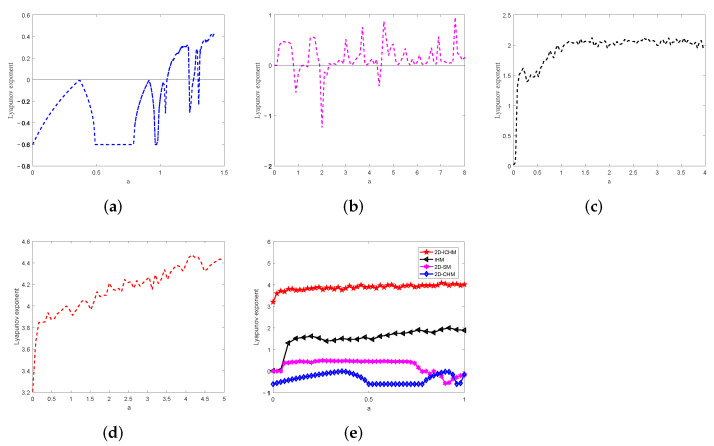
The largest LE. (**a**) 2D-CHM with b=0.3, (**b**) 2D-SM with b=3.8, (**c**) IHM with b=0.3 and r=0.1, (**d**) 2D-ICHM with b=5, (**e**) comparison of four maps.

The LEs of 2D-CHM, 2D-SM, IHM, and 2D-ICHM are calculated by QR decomposition algorithm, and Figure 3 plots their largest LEs. The figures are obtained by changing the parameter *a* when other parameters are fixed. A comparison on the largest LEs of the above four chaotic systems is given in Figure 3e. It is noted from this that 2D-ICHM has a larger and continuous positive LE value. Thus, 2D-ICHM has a more continuous chaotic range, which means it is suitable for image encryption systems.

(3) Approximate entropy

The complexity of nonlinear time series can be evaluated by the approximate entropy (ApEn), which increases with the increase of ApEn value. The calculation process of the ApEn is shown as follows [41]:

**Step 1:** Given a time series $x\left(1\right),x\left(2\right),\cdot$ $\cdot \cdot ,x\left(N\right)$, divide them into *m*-dimensional vectors

(5) X(i)=[x(i),x(i+1),⋯,x(i+m−1)],

where i=1,2,3,⋯,N−m+1.

**Step 2:** Measure the distance between X(i) and X(j) by

(6) $$d\left(i,j\right)=\max_{k=0,1,\cdots ,m-1}\left[\left|x(i+k)-x(j+k)\right|\right],$$

where i=1,2,3,⋯,N−m+1, j=1,2,3,⋯,N−m+1.

**Step 3:** Set a threshold value r(r>0), define for each *i*, 1≤i≤N−m+1,

(7) Cim(r)=numberofjsuchthatd(i,j)<rnumberofjsuchthatd(i,j)<rN−m+1N−m+1,

where j=1,2,3,⋯,N−m+1.

**Step 4:** Denote the mean of logarithm of $C_{i}^{m}\left(r\right)$ as $\varphi^{m}\left(r\right)$ and we have

(8) $$\varphi^{m}\left(r\right)=\frac{1}{N-m+1}\sum_{i=1}^{N-m+1}\ln C_{i}^{m}\left(r\right).$$

**Step 5:** Change the dimension *m* to m+1 and repeating step 1 to step 4, the ApEn is

(9) $$ApEn\left(m,r\right)=\lim_{N\to \infty }\left[\varphi^{m}\left(r\right)-\varphi^{m+1}\left(r\right)\right].$$

In practical terms, the length of the data sequence is bounded. Therefore, the ApEn algorithm is changed into

(10) $$ApEn\left(m,r,N\right)=\varphi^{m}\left(r\right)-\varphi^{m+1}\left(r\right).$$

**Figure 4:**
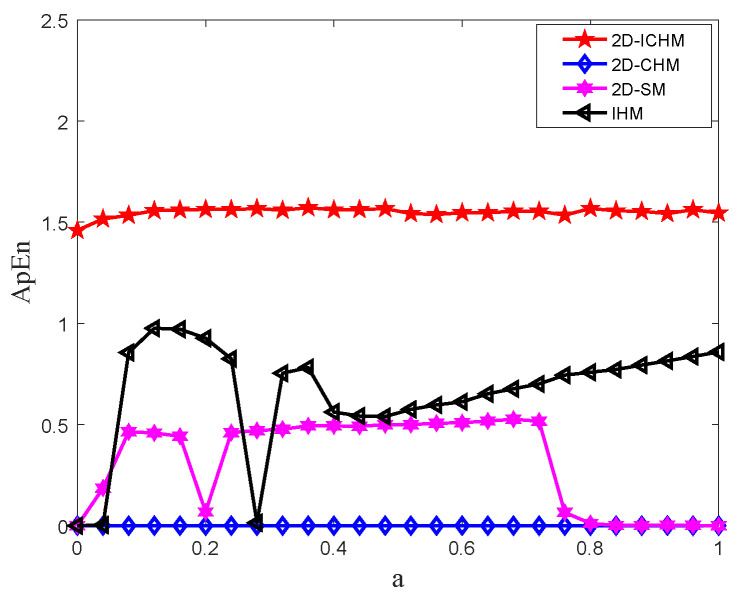
The ApEn comparison among the 2D-ICHM (b=5), 2D-CHM (b=0.3), 2D-SM (b=3.8, r=0.1), and IHM (b=0.3).

In order to keep the correlation between ApEn and N to a minimum, Pincus found that parameters can be set to m=2 and $r\in \left[0.1S\;D\left(x\right),0.2S\;D\left(x\right)\right]$, $S\;D\left(x\right)$ is the standard deviation of *x* [42]. Using the above algorithm, the ApEn values of the 2D-CHM, 2D-SM, IHM, and 2D-ICHM are shown in Figure 4. As shown in Figure 4, 2D-ICHM has a higher ApEn value; therefore, the output time series of 2D-ICHM has higher complexity.

(4) NIST SP800-22 test

The level of security of an image encryption system depends heavily on the randomness of the pseudorandom number sequence. NIST SP800-22 test [43] can be used to evaluate the random characteristics of binary bit sequences. The NIST SP800-22 test provides 15 test methods, including frequency test, run test, approximate entropy test, random excursions test, etc. Each test calculates a random value to determine whether the binary sequence is random. If p_value>0.01, the binary sequence is considered to be random, and the larger the p_value, the better the randomness. The SP800-22 test recommends that the length of the binary sequence tested is from $10^{3}$ to $10^{7}$. Therefore, the test binary sequence we used is $10^{6}$ in length. As we can see from Table 1, all the calculated p_value are larger than 0.01. Therefore, the 2D-ICHM has passed all the random tests, which shows that the 2D-ICHM is more suitable for image encryption.

(5) 0–1 test

G. A. Gottwald and I. Melbourne proposed a reliable and effective binary test method for checking whether the dynamical system is chaos, which is called the “0–1 test” [44]. It can be described as

**Step 1:** For a time series x(j)(j=1,2,⋯,N), the definition of translation variables is

(11) $$\left\{\begin{array}{c} p_{c}\left(n\right)=\sum_{j=1}^{n}x(j)\cos\left(jc\right),\\ q_{c}\left(n\right)=\sum_{j=1}^{n}x(j)\sin\left(jc\right), \end{array}\right.$$

where c∈(0,π) and n=1,2,⋯,N.

**Step 2:** In order to measure the diffusive (or nondiffusive) behavior of $p_{c}$ and $q_{c}$, the mean square displacement defined as

(12) $$M_{c}\left(n\right)=\lim_{N\to \infty }\frac{1}{N}\sum_{j=1}^{N}\left\{{\left[p_{c}\left(j+n\right)-p_{c}\left(j\right)\right]}^{2}+{\left[q_{c}\left(j+n\right)-q_{c}\left(j\right)\right]}^{2}\right\},$$

where $n\leq N_{0}<<N$. In practice, $N_{0}=round\left(N/10\right)$.

**Step 3:** Define the modified mean square displacement $D_{c}\left(n\right)$ as

(13) $$D_{c}\left(n\right)=M_{c}\left(n\right)-V_{osc}\left(c,n\right),$$

where $V_{osc}\left(c,n\right)={\left[\lim_{N\to \infty }\frac{1}{N}\sum_{j=1}^{N}x(j)\right]}^{2}\frac{1-\cos\left(nc\right)}{1-\cos\left(c\right)}$.

**Step 4:** Define the vectors and the correlation coefficient

(14) $$\left\{\begin{array}{c} \Delta =(D_{c}\left(1\right),D_{c}\left(2\right),\cdots ,D_{c}\left(N_{0}\right)),\\ K_{c}=corr\left(\xi ,\Delta \right)\in \left[-1,1\right], \end{array}\right.$$

where $\xi =1,2,\cdots ,N_{0}$. $K_{c}\approx 0$ indicates regular behaviour, while $K_{c}\approx 1$ indicates chaotic behaviour.

**Figure 5:**
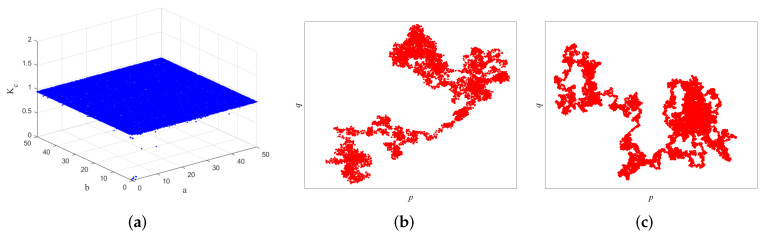
0–1 test results. (**a**) Plot of $K_{c}$ versus *a* and *b*, (**b**) (p,q) plane of the *x* sequence with $\left(a,b\right)\;=\;\left(5,5\right)$, (**c**) (p,q) plane of the *y* sequence with $\left(a,b\right)\;=\;\left(5,5\right)$.

Figure 5 shows the 0–1 test results of 2D-ICHM with c=2, and initial values $\left(x\left(0\right),y\left(0\right)\right)=\left(0.3,0.3\right)$. As shown in Figure 5a, $K_{c}$ is very close to 1, illustrating that the 2D-ICHM has significant chaotic behavior. In addition, The (p,q) plane also can intuitively reflect whether the dynamic system is chaotic or not. When the trajectory of the (p,q) plane is bounded motion, the dynamical system is regular, and when the trajectory of the (p,q) plane is Brownian-like motion, the dynamical system is chaotic. The (p,q) planes of the 2D-ICHM are shown in Figure 5b,c. It can be seen that the trajectories of 2D-ICHM are similar to Brownian motion. This means that the 2D-ICHM is a chaotic dynamic system.

## 4. Relevant Knowledge

### 4.1. Bit-Plane Decomposition

The recombined plane of binary pixel values at the same bit positions of a grayscale image is called the bit plane. The grayscale image P={p(i,j)} is decomposed into eight binary bit planes $P_{k}=\left\{p_{k}\left(i,j\right)\right\}\left(k=1,2,\cdots ,8\right)$ [45], given by

(15) $$P=\sum_{k=1}^{8}P_{k}2^{k-1}=P_{1}2^{0}+P_{2}2^{1}+\cdots +P_{8}2^{7}.$$

**Figure 6:**
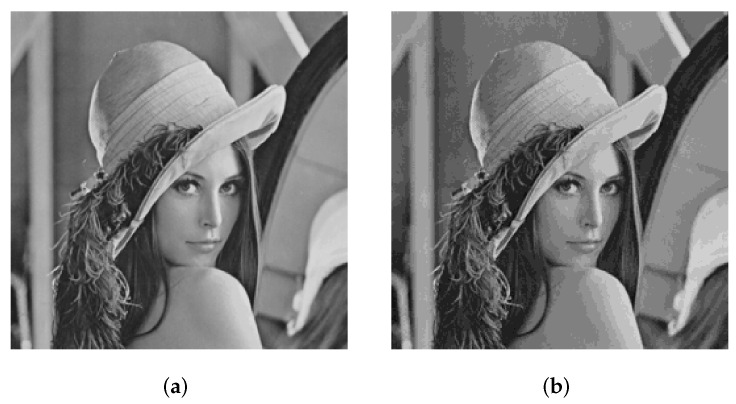
Lena and composite image of high four bit planes. (**a**) Lena, (**b**) composite image of high four bit planes.

**Figure 7:**
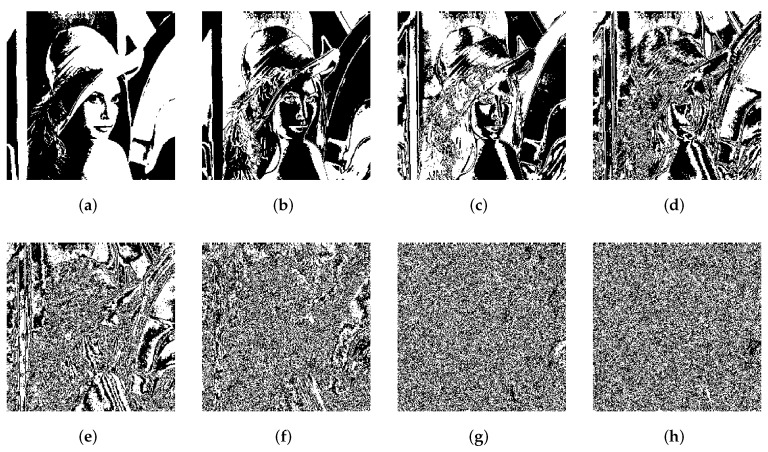
The corresponding eight bit planes of Lena. (**a**) $P_{8}$, (**b**) $P_{7}$, (**c**) $P_{6}$, (**d**) $P_{5}$, (**e**) $P_{4}$, (**f**) $P_{3}$, (**g**) $P_{2}$, and (**h**) $P_{1}$.

Figure 6a is a grayscale image “Lena” of size 256 × 256. The eight binary planes of “Lena” are shown in Figure 7a–h. The higher bit plane contains more information, among which the high four bit planes contain more than 94% of information in the original image [46]. A composite image of high four bit planes is shown in Figure 6b, which retains the vast majority of the original image.

### 4.2. Integer Wavelet Transform

**Figure 8:**
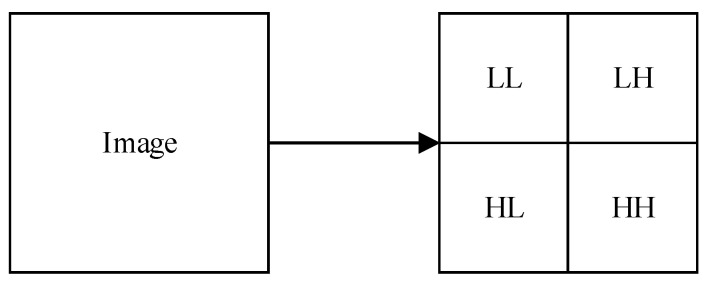
IWT operations.

Wavelet transform links the time domain and frequency domain of the image. The IWT was proposed by Swelden and Daubechies in 1998 [47]. Compared with the traditional wavelet transform, the IWT has obvious advantages, e.g., low computational complexity, no edge effect, and complete reversibility. The image can be decomposed into four bands LL, LH, HL, and HH using IWT (see Figure 8). Most of the detailed information in the image is concentrated in the low frequency band LL [48].

### 4.3. DNA Sequence Operations

DNA sequence operations consist of two components: DNA encoding/decoding and DNA computation.

(1) DNA encoding and decoding rules

The DNA sequence of biology contains four nucleic acid bases i.e., A (Adenine), C (Cytosine), G (Guanine), and T (Thymine), where A and T, G, and C are complementary, respectively [49]. In binary computing, 0 and 1 are complementary, so the binary digits 00 and 11 are complementary, as well as 01 and 10. The binary digits 00, 01, 10, and 11 can be encoded as the four bases A, T, C, and G. There are 24 kinds of coding rules, while only eight coding rules are capable of meeting the Watson-Crick complement rule, as listed in Table 2. A pixel value denoted by eight bits can be encoded as a DNA sequence containing four bases. For example, a decimal pixel value is 150, and its corresponding binary is [10010110]. Different coding rules yield different combinations of bases. If we use Rule 3, [10010110] is encoded as [TAAT]. Decoding is the inverse process of encoding. The inverse of Rule 3 can be used to decode [TAAT] into [10010110].

(2) DNA computation

The computation of DNA sequences includes DNA addition, subtraction, and XOR operations, where DNA addition and DNA subtraction operations are reciprocal. These three DNA computations are all used in this paper. The eight different DNA coding rules in Table 2 correspond to eight different DNA addition, subtraction and XOR operations. In this paper, we use the coding Rule 4, whose corresponding DNA addition and XOR operations are shown in Table 3.

## 5. The Proposed Image Encryption Algorithm

### 5.1. Generating Chaotic Sequences

In order to enhance the correlation of the proposed algorithm and the plain image. the system parameters and initial values of the 2D-ICHM are generated by the SHA-512 hash values of the plain image. The process of generating chaotic sequences is specified as follows.

**Step 1:** The SHA-512 hash values of the plain image are divided into 64 8-bit blocks: $K=\left[k_{1},k_{2},\cdots ,k_{64}\right]$. The parameters $s_{1},s_{2},s_{3},s_{4},s_{5},s_{6}$ can be calculated by

(16) $$\left\{\begin{array}{c} s_{1}=\frac{k_{1}+k_{2}+\cdots +k_{8}}{8\times 2^{8}},\;\\ s_{2}=\frac{k_{9}⊕k_{10}⊕\cdots ⊕k_{16}}{2^{8}},\;\\ s_{3}=\frac{k_{17}⊕k_{18}⊕\cdots ⊕k_{24}}{2^{8}},\;\\ s_{4}=\frac{k_{25}⊕k_{26}⊕\cdots ⊕k_{32}}{2^{8}},\;\\ s_{5}=\frac{\left(k_{33}+k_{34}\right)⊕\left(k_{35}+k_{36}\right)⊕\cdots ⊕\left(k_{47}+k_{48}\right)}{2\times 2^{8}},\;\\ s_{6}=\frac{\left(k_{49}⊕k_{50}\right)+\left(k_{51}⊕k_{52}\right)+\cdots +\left(k_{63}⊕k_{64}\right)}{8\times 2^{8}}, \end{array}\right.$$

where x⊕y is the bitwise XOR operator. The system parameters $a_{0},b_{0}$ and initial values $x_{0},y_{0}$ of 2D-ICHM are calculated as follows.

(17) a0=mod((s1+s2+s3)×108,256)mod((s1+s2+s3)×108,256)255255+v1,b0=mod((s2+s3+s4)×108,256)mod((s2+s3+s4)×108,256)255255+v2,x0=mod((s3+s4+s5)×108,256)mod((s3+s4+s5)×108,256)255255+v3,y0=mod((s4+s5+s6)×108,256)mod((s4+s5+s6)×108,256)255255+v4,

where $v_{1},v_{2},v_{3},v_{4}$ are real numbers. The *K*, $v_{1}$, $v_{2}$, $v_{3}$ and $v_{4}$ are secret keys.

**Step 2:** To eliminate the transient effect and improve security of the system, 2D-ICHM is performed with $N_{0}$ pre-iterations. Then it is iterated M×N times, where *M* and *N* represent the width and height of the plain image, respectively. We use *i* to represent the index of the number of iterations. After each iteration, state values X(i),Y(i) are stored in the sequence X,Y, respectively.

**Step 3:** The two chaotic sequences $X^{1},Y^{1}$ are calculated by

(18) $$\left\{\begin{array}{c} X^{1}\left(i\right)=\;\;\mathrm{mod}\;\;\left(\left\lfloor \left(\left|X(i)\right|-\left\lfloor \left|X(i)\right|\right\rfloor \right)\times 2^{16}\right\rfloor ,256\right),\;\\ Y^{1}\left(i\right)=\;\;\mathrm{mod}\;\;\left(\left\lfloor \left(\left|Y(i)\right|-\left\lfloor \left|Y(i)\right|\right\rfloor \right)\times 2^{16}\right\rfloor ,256\right), \end{array}\right.$$

where $\left\lfloor \cdot \right\rfloor$ denotes the round-down operation, and i=1,2,⋯,M×N.

### 5.2. Encryption Process

The encryption process is as follows. The process of high bit planes diffusion in the space domain corresponds to Steps 2–3, the process of scrambling operation in the frequency domain corresponds to Steps 4–7, and the process of DNA computing and bidirectional diffusion in the spatial domain corresponds to Steps 8–10.

**Step 1:** The plain image *P* of size M×N is decomposed into eight binary bit planes $P_{1},P_{2},\cdots ,P_{8}$.

**Step 2:** Arrange the high bit planes $P_{i}$$\left(i=5,6,7,8\right)$ into binary vectors $P_{i}^{\prime }$$\left(i=5,6,7,8\right)$ from top to down row by row. Take the first MNMN88 terms of the chaotic sequence $X^{1}$ and convert it into the binary sequence $H_{1}$.

**Step 3:** The new binary vectors $P_{i}^{\prime \prime }\left(i=5,6,7,8\right)$ are generated by the diffusion operation of Equation (19). The vectors $P_{i}^{\prime \prime }\left(i=5,6,7,8\right)$ are transformed into the bit planes ${\hat{P}}_{i}$ $\left(i=5,6,7,8\right)$ according to the top to down and left to right rules.

(19) $$\left\{\begin{array}{c} P_{8}^{\prime \prime }=bitxor\left(P_{8}^{\prime },H_{1}\right),\;\\ P_{7}^{\prime \prime }=bitxor\left(P_{7}^{\prime },{\hat{P}}_{8}\right),\;\\ P_{6}^{\prime \prime }=bitxor\left(P_{6}^{\prime },{\hat{P}}_{7}\right),\;\\ P_{5}^{\prime \prime }=bitxor\left(P_{5}^{\prime },{\hat{P}}_{6}\right), \end{array}\right.$$

where $bitxor\left(x,y\right)$ represents bit by bit XOR operations. The intermediate cipher image $Q_{1}$ is obtained using Equation (20).

(20) $$Q_{1}=P_{1}2^{0}+P_{2}2^{1}+P_{3}2^{2}+P_{4}2^{3}+{\hat{P}}_{5}2^{4}+{\hat{P}}_{6}2^{5}+{\hat{P}}_{7}2^{6}+{\hat{P}}_{8}2^{7}.$$

**Figure 9:**
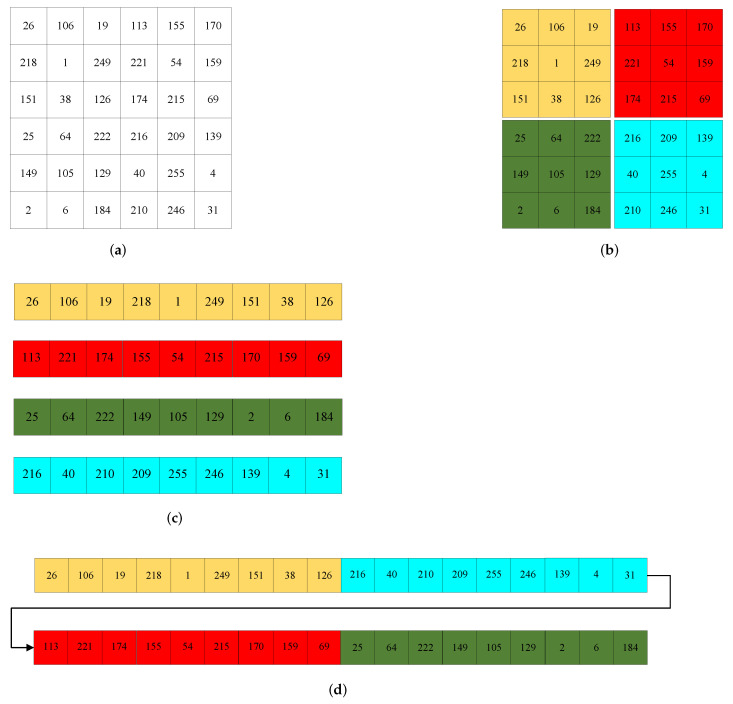
Example of Chunking—Arrangement—Combination. (**a**) 6×6 matrix, (**b**) chunking operations, (**c**) arrangements of sub-blocks, and (**d**) combination of vectors.

**Step 4:** The IWT is applied to $Q_{1}$ to obtain the bands LL, LH, HL and HH of each size MM22×NN22. To visualize the Chunking-Arrangement-Combination operation, an example is provided in Figure 9 (M=12,N=12).

**Step 5:**LL is divided into 4 sub-blocks of size m×n (see Figure 9b, m=3,n=3). Convert each sub-block to a vector of length m×n by arranging the first column sub-blocks from left to right row by row and the second column sub-blocks from top to down column by column (see Figure 9c). After that, sub-vectors are recombined into a vector $Z_{1}$ of length 4×m×n according to the combination method of Figure 9d.

**Step 6:** Using the method in Step 5 to convert LH, HL and HH to vectors $Z_{2}$, $Z_{3}$ and $Z_{4}$, respectively. Take the first MNMN44 terms of the chaotic sequence $X^{1}$ to obtain the sequence $X^{2}$. By arranging the sequence $X^{2}$ in ascending order, the index sequence *I* is obtained.

**Step 7:** The vectors $Z^{1}$, $Z^{2}$, $Z^{3}$ and $Z^{4}$ are obtained by using the global scrambling operation of Equation (21).

(21) $$Z^{i}\left(j\right)=Z_{i}\left(I\left(j\right)\right),$$

where i=1,2,3,4 and j=1,2,⋯,M×NM×N44. Then $Z^{1}$, $Z^{2}$, $Z^{3}$ and $Z^{4}$ are transformed into matrices $L\;L^{1}$, $L\;H^{1}$, $H\;L^{1}$ and $H\;H^{1}$ according to the top to down and left to right rules. The intermediate cipher $Q_{2}$ is obtained by applying inverse IWT of $L\;L^{1}$, $L\;H^{1}$, $H\;L^{1}$ and $H\;H^{1}$.

**Step 8:** Arrange $Q_{2}$ into binary vectors $Q_{2}^{\prime }$ from top to down row by row, and convert chaotic sequence $Y^{1}$ into binary sequence $H_{2}$. Convert $Q_{2}^{\prime },$ $H_{1},H_{2}$ into DNA sequences ${\hat{Q}}_{2}^{\prime },H_{1}^{1},H_{2}^{1}$ by DNA coding Rule 4 in Table 2. Then the DNA addition and XOR operations (see Table 3) are performed on the above DNA sequences using Equation (22) to obtain the sequence $H_{3}$.

(22) $$H_{3}=DNA\_xor\left(DNA\_add\left({\hat{Q}}_{2}^{\prime },H_{1}^{1}\right),H_{2}^{1}\right).$$

**Step 9:** The inverse of DNA coding Rule 3 is used to decode $H_{3}$ to obtain the binary sequence $Q_{3}^{\prime }$.

**Step 10:**$Q_{3}^{\prime }$ is converted to the decimal sequence $Q_{3}$. Then we use the bidirectional diffusion processing of Equation (23) to obtain the sequence $Q_{4}$.

(23) $$\left\{\begin{array}{c} E\left(i\right)=E\left(i-1\right)\times X^{1}\left(i\right)\times Q_{3}\left(i\right),\;\\ Q_{4}\left(j\right)=Q_{4}\left(j+1\right)\times Y^{1}\left(j\right)\times E\left(j\right), \end{array}\right.$$

where i=1,2,⋯,MN, j=MN−1,MN−2,⋯,1, “×” denotes GF(257) field multiplication, $E\left(0\right)$ and $Q_{4}\left(0\right)$ are positive integers and take values in the range 0 to 255, $Q_{4}\left(MN\right)=Q_{4}\left(0\right)\times Y^{1}\left(MN\right)\times E\left(MN\right)$. $E\left(0\right)$ and $Q_{4}\left(0\right)$ are secret keys.

**Step 11:** The $Q_{4}$ is transformed into the final encrypted image *C* according to the top to down and left to right rules.

**Figure 10:**
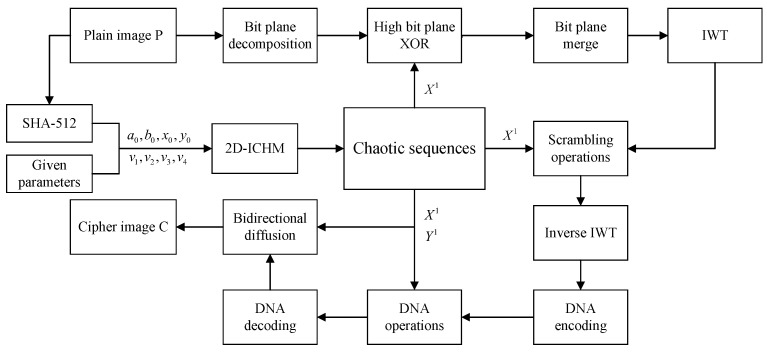
Encryption flow chart.

**Figure 11:**
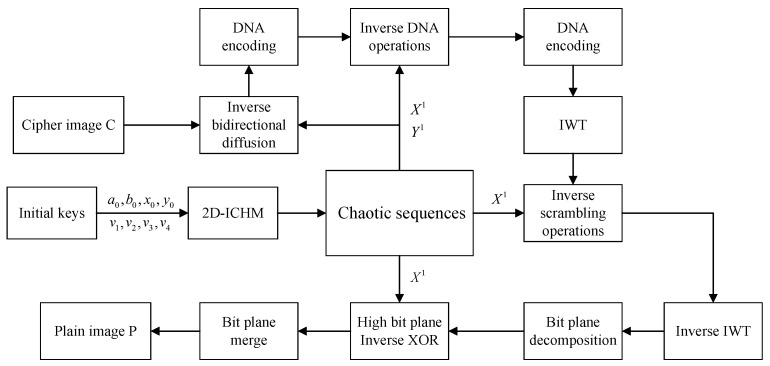
Decryption flow chart.

The encryption flow chart of the proposed algorithm is shown in Figure 10. Decryption can be completed by performing the reverse operation of encryption. The decryption flow chart is shown in Figure 11.

## 6. Simulation Results

The experimental environment is Intel(R) Core(TM) i5-9300HF CPU processor operating at 2.4 GHz, 8 GB of RAM, and a Microsoft Windows 10 operation system. We use Matlab 2016b to execute encryption and decryption programs. The experimental images are chosen from the CVG-UGR and USC-SIPI image databases. The parameters used in this paper are as follows: $v_{1}=80$, $v_{2}=20$, $v_{3}=2,v_{4}=2$, $E\left(0\right)=20$, $Q_{4}\left(0\right)=20$, and the size of sub-blocks m×n=64×64. Four different 256×256 grayscale images “Lena”, “Peppers”, “5.1.10”, and “5.1.11” are used as plain images.

**Figure 12:**
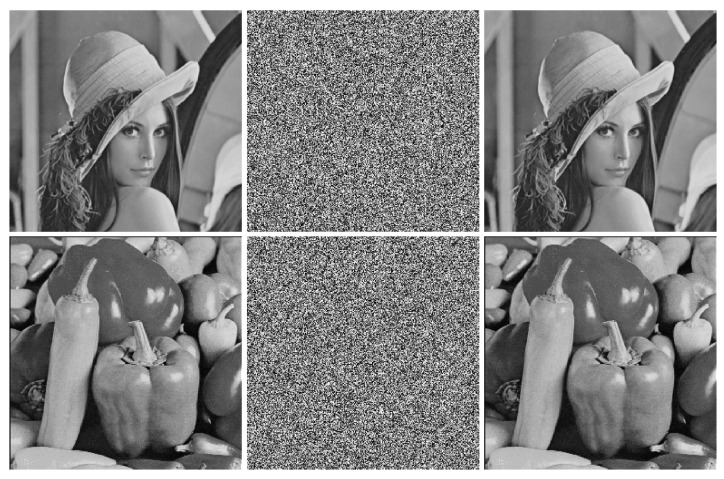

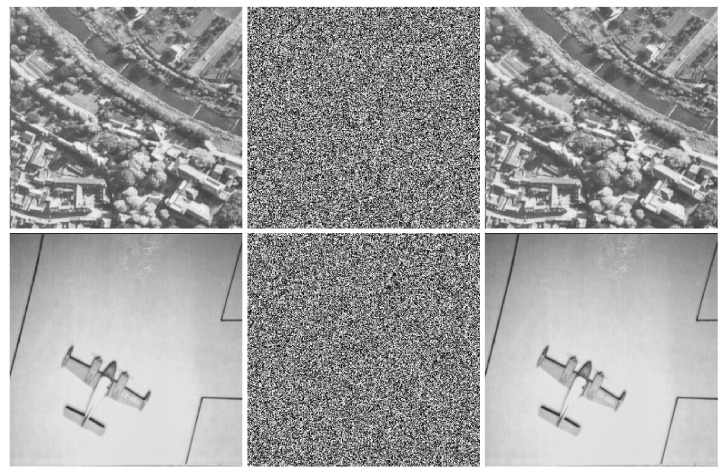
The simulation results of the proposed image encryption algorithm. The first column: plain images; the second column: encrypted images; the third column: decrypted images.

The results of encryption and decryption are displayed in Figure 12. As can be seen, the cipher images are noise-like. It means that we cannot get useful information about the plain images from the cipher images. Furthermore, the decrypted images are identical to the plain images in visual respects. Thus, the proposed image encryption algorithm has excellent encryption and decryption effects.

## 7. Security Analysis

In this section, we evaluate the security performance of the proposed algorithm through the analysis of key space, key sensitivity, histogram, correlation, information entropy, differential attack, chosen/known-plaintext attack, cropping attack, and noise attack.

### 7.1. Key Space Analysis

To counter brute force attacks, we should expand the key space of the algorithm as much as possible. The literature [50] stated that the key space of a secure encryption algorithm should be larger than $2^{100}$. The secret key of the proposed algorithm includes three subkeys: (1) 512-bit hash value *K* of the plain image; (2) the given parameters $v_{1},v_{2},v_{3}$, and $v_{4}$; (3) the positive integers $E\left(0\right)$ and $Q_{4}\left(0\right)$. Suppose the operational precision of the computer is $10^{-14}$; the key space of the proposed algorithm is $2^{512}\times 10^{14\times 4}\times 256\times 256>2^{714}$, which is much larger than $2^{100}$. The results compared with other algorithms are listed in Table 4. From Table 4, it is obvious that our key space is larger, which means that the proposed algorithm is resistant to brute force attacks.

### 7.2. Key Sensitivity Analysis

A secure image encryption system should show a high sensitivity to the key. The key sensitivity can be considered in two aspects.

**Figure 13:**
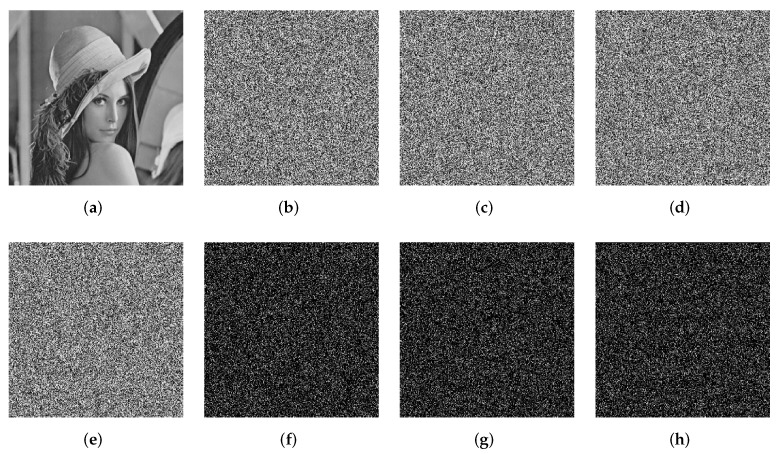
The key sensitivity analysis results of the encryption process. (**a**) Plain image “Lena”, (**b**) cipher image *C* with correct keys, (**c**) cipher image $C_{1}$ with $K_{1}$, (**d**) cipher image $C_{2}$ with $v_{1}^{\prime }=v_{1}+10^{-14}$, (**e**) cipher image $C_{3}$ with $v_{3}^{\prime }=v_{3}-10^{-14}$, (**f**) subtraction image $S_{1}=\left|C_{1}-C\right|$, (**g**) subtraction image $S_{2}=\left|C_{2}-C\right|$, and (**h**) subtraction image $S_{3}=\left|C_{3}-C\right|$.

In the encryption process, using two keys with a tiny difference to encrypt the same plain image, the two encrypted images should be completely different. Take “Lena” as test image. The key sensitivity analysis results of the encryption process are shown in Figure 13, where $K_{1}$ is obtained by changing the 1st bit of *K* from 1 to 0.The cipher images $C_{1}$, $C_{2}$, and $C_{3}$ (Figure 13c–e) are obtained by using slightly different keys (A subkey is changed while the other subkeys remain unchanged). The subtraction images $S_{1}$, $S_{2}$, and $S_{3}$ (Figure 13f–h) with noiselike textures indicate that $C_{1}$, $C_{2}$, and $C_{3}$ are totally different from *C*.

**Figure 14:**
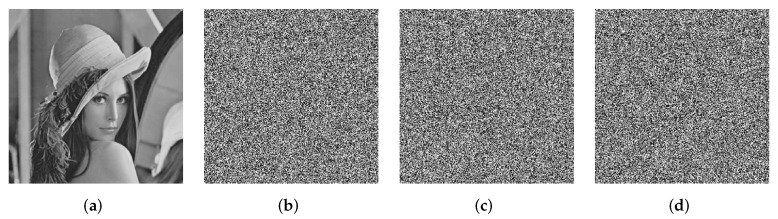
The key sensitivity analysis results of the decryption process. (**a**) Decrypted image *D* with correct keys, (**b**) decrypted image $D_{1}$ with $K_{1}$, (**c**) decrypted image $D_{2}$ with $v_{1}^{\prime }=v_{1}+10^{-14}$, and (**d**) decrypted image $D_{3}$ with $v_{3}^{\prime }=v_{3}-10^{-14}$.

In the decryption process, the plain image can only be decrypted correctly when the correct secret key is used. The key sensitivity analysis results of the decryption process are shown in Figure 14. It can be seen that when the decryption keys with a tiny difference are used, the decrypted images become noise images. The decrypted images are totally different from the correct plain images.

To quantitatively evaluate the key sensitivity of the proposed algorithm, the number of pixels change rate (NPCR) and unified average changing intensity (UACI) are adopted. For two random 8-bit noise images, the ideal values of NPCR and UACI are 99.61% and 33.46% [55]. The formula is defined as follows.

(24) $$\left\{\begin{array}{c} NPCR=\frac{\sum_{i=1}^{M}\sum_{j=1}^{N}D\left(i,j\right)}{M\;\times \;N}\times 100\%,\;\\ UACI=\frac{\sum_{i=1}^{M}\sum_{j=1}^{N}\left|C_{1}\left(i,j\right)\;-\;C_{2}\left(i,j\right)\right|}{M\;\times \;N\;\times \;255}\times 100\%, \end{array}\right.$$

where

(25) $$D\left(i,j\right)=\left\{\begin{array}{c} 1,C_{1}\left(i,j\right)\;\neq \;C_{2}\left(i,j\right),\\ 0,C_{1}\left(i,j\right)\;=\;C_{2}\left(i,j\right), \end{array}\right.$$

$C_{1},C_{2}$ represents two different cipher images, M × N represents the size of image.

The calculated values of NPCR and UACI between the cipher image *C* (Figure 13b) and the cipher images *C*, $C_{1}$, $C_{2}$, and $C_{3}$ (Figure 13b–e) are listed in Table 5. It can be seen that the values of NPCR and UACI are close to the ideal values. This means that when slightly different keys are used in the encryption process, the cipher images obtained are totally different. Between a random noise image and a determinate Lena image, the ideal value of NPCR is 99.61% and the ideal value of UACI is 28.62% [56]. The calculated values of NPCR and UACI between the decrypted image *D* (Figure 14a) and the decrypted images *D*, $D_{1}$, $D_{2}$, and $D_{3}$ (Figure 14a–d) are listed in Table 6. It is clear that the values of NPCR and UACI are close to the ideal values. This means that when slightly different decryption keys are used in the decryption process, the decrypted images obtained are totally different. Therefore, the proposed encryption algorithm has a high sensitivity to the secret key.

### 7.3. Histogram Analysis

**Figure 15:**
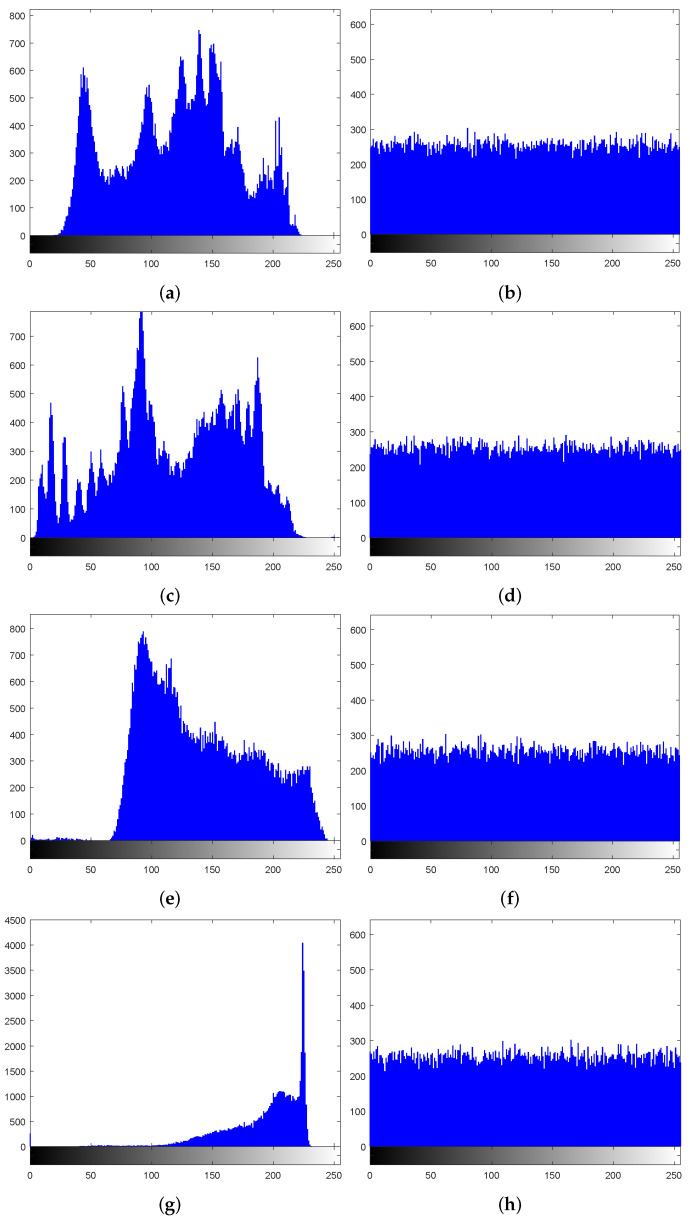
Histogram results. Plain image: (**a**) Lena, (**c**) Peppers, (**e**) 5.1.10, and (**g**) 5.1.11; cipher image: (**b**) Lena, (**d**) Peppers, (**f**) 5.1.10, and (**h**) 5.1.11.

The distribution of image pixel values can be reflected by the image histogram. If the histogram of a cipher image is flat, information of the plain image is excellently hidden. Figure 15 shows the histograms of the images before and after encryption. It can be seen that the histograms of encrypted images become relatively flat. Therefore, the proposed algorithm can effectively resist statistical attacks.

The chi-square test can be used to quantitatively analyze the uniformity of the histogram, which is defined by Equation (26).

(26) $$\chi^{2}=\sum_{i=0}^{255}\frac{\left(f_{i}-g\right)}{g},$$

where g=M×NM×N256256, and $f_{i}$ is the occurrence frequency of the pixels whose value is *i*. Given a significant level α=0.05, if $\chi_{0.05}^{2}<293.2478$, the chi-square test is passed [57]. Table 7 shows that the calculated chi-square values for all cipher images are less than 293.2478. Therefore, all the cipher images encrypted by the proposed algorithm have passed the chi-square test, which means that the proposed algorithm can resist statistical attacks.

### 7.4. Correlation Analysis of Adjacent Pixels

**Figure 16:**
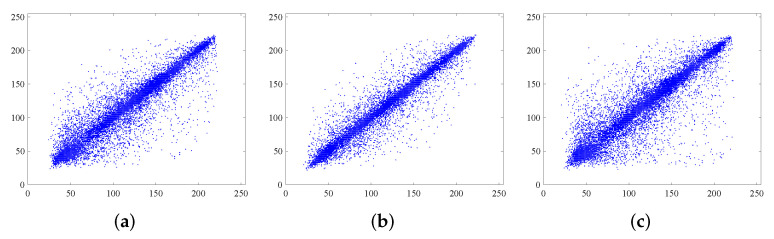

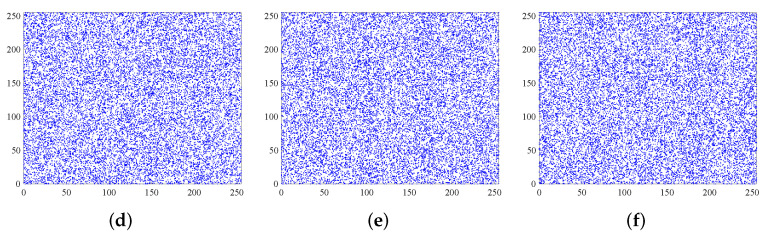
Correlation analysis of Lena before and after encryption. (**a**,**d**) horizontally adjacent, (**b**,**e**) vertically adjacent, (**c**,**f**) diagonally adjacent.

The plain image with effective information has a strong correlation between adjacent pixels. The ideal encryption algorithm can eliminate the correlation of adjacent pixels to resist statistical attacks. To ensure the reliability of the experiment, 20,000 pairs of pixels are randomly selected to test the correlation in horizontal, vertical, and diagonal directions. As shown from Figure 16, the adjacent pixel distribution of the plain image is relatively concentrated, whereas the adjacent pixel distribution of the cipher image is noiselike. This means that the correlation of the plain image is greatly reduced. To quantitatively describe the correlation, the correlation coefficient is calculated as follows.

(27) $$\left\{\begin{array}{c} E\left(x\right)=\frac{1}{N}\sum_{i=1}^{N}x_{i},\\ cov\left(x,y\right)=\frac{1}{N}\sum_{i=1}^{N}\left(x_{i}-E\left(x\right)\right)\left(y_{i}-E\left(y\right)\right),\\ D\left(x\right)=\frac{1}{N}\sum_{i=1}^{N}{\left(x_{i}-E\left(x\right)\right)}^{2},\;\\ r_{xy}=\frac{cov\left(x,y\right)}{\sqrt{D\left(x\right)}\sqrt{D\left(y\right)}}. \end{array}\right.$$

The calculated correlation coefficients are shown in Table 8. It can be seen that the correlation coefficients of the cipher images have been greatly reduced, close to 0. The results compared with other algorithms as shown in Table 9. As can be seen, the correlation coefficients of Lena for the proposed algorithm are smaller in all three directions compared with [6,54], and the proposed algorithm has great advantages in the horizontal and diagonal directions compared with [16,51,52], and the proposed algorithm has certain advantages in the horizontal direction compared with [53]. The above results show that the proposed algorithm can effectively remove the correlation of adjacent pixels, so it provides a high level of security to resist statistical attacks.

### 7.5. Information Entropy

Information entropy is an important indicator to describe the uncertainty of image information, which quantifies the distribution of the image’s grayscale values [17]. Generally speaking, the higher the information entropy value, the higher the degree of disorder in the image [52]. The formula of information entropy is as follows.

(28) $$H\left(s\right)=-\sum_{i=1}^{L}p\left(x_{i}\right)\log_{2}p\left(x_{i}\right),$$

where *L* is the grayscale grade of the image, and $p\left(x_{i}\right)$ is the probability of the grayscale value $x_{i}$.

For 8-bit noise type grayscale images, the ideal value of information entropy is 8. The information entropy of different plain images and their corresponding cipher images are listed in Table 10. As can be seen, values of the information entropy of all encrypted images are close to 8. Table 11 lists the comparison results with other algorithms for Lena. It is obvious that the proposed algorithm owns a larger information entropy compared with [6,16,51,52,53,54], which means the cipher images encrypted by the proposed algorithm have a stronger randomness. Thus, the proposed algorithm can resist statistical attacks based on entropy.

### 7.6. Differential Attack Analysis

A secure image encryption algorithm should have excellent capability to resist differential attacks. Attackers can encrypt two slightly different plain images using the same algorithm, and then try to establish a link between the plain and cipher images by comparing the two encrypted images. The NPCR and UACI are able to evaluate whether encryption algorithms can resist differential attacks. The study in the literature [58] pointed out that the algorithm is resistant to differential attacks when the $NPCR>N_{\alpha }$ and $U_{\alpha }^{-}<UACI<U_{\alpha }^{+}$, where $N_{\alpha }$, $U_{\alpha }^{-}$, $U_{\alpha }^{+}$ are the critical values and α is the significance level. The critical values for images of size 256×256 are listed in Table 12.

To test the performance of the proposed algorithm against differential attacks, we randomly change a pixel value of the plain image to obtain the modified plain image. Subsequently, the two plain images are encrypted by the proposed algorithm to get the cipher images. The test is performed over 100 times with different test images. The mean values of the test results are listed in Table 13 and Table 14, respectively. It can be seen that the proposed algorithm passes the test and is resistant to differential attacks. Table 15 lists a comparison of the NPCR and UACI values of Lena for different encryption algorithms. As can be seen, the NPCR and UACI values of Lena for the proposed algorithm are closer to the ideal value compared with [16,54], and the proposed algorithm has some merits compared with [51,53]. Thus, the proposed algorithm is capable of resisting differential attacks.

### 7.7. Chosen/Known-Plaintext Attack Analysis

Chosen-plaintext and known-plaintext attacks are prevalent and high-threat types of attacks. The literature [59] indicated that an encryption algorithm with the capability to resist chosen-plaintext attacks can also resist known-plaintext attacks. Therefore, we only consider resisting chosen-plaintext attacks.

In the proposed algorithm, we exploit the SHA-512 hash values of the plain image to generate the system parameters and initial values of the chaotic system, making the proposed algorithm highly sensitive to the plain image. Thus, when attackers use the proposed algorithm to encrypt slightly changed plain images, the encryption result obtained is totally different. Attackers cannot gain the desired information using special images. Furthermore, we perform bit-level exclusive-or operations between different bit-planes. Attackers are incapable of using special images to simplify the diffusion process.

**Figure 17:**
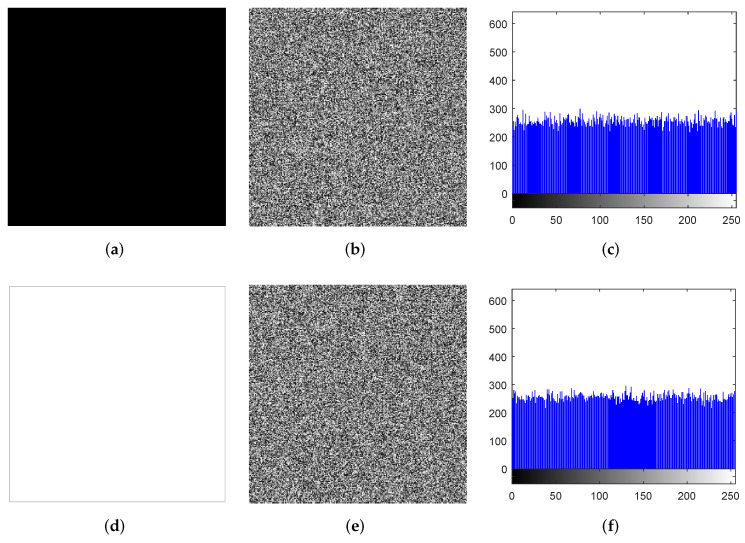
Experimental results of “all-black” and “all-white”. (**a**) “all-black”, (**b**) encryption “all-black”, (**c**) histogram of encryption “all-black”, (**d**) “all-white”, (**e**) encryption “all-white”, (**f**) histogram of encryption “all-white”.

Attackers often use all-black or all-white plain images as special images to attack encryption algorithms, since such special images can disable the scrambling process [55]. We leverage the all-black and all-white plain images with the size 256×256 in the experiment, and the results are shown in Figure 17. It can be seen that the cipher images are noiselike images, and the histograms of the cipher images are quite flat. Attackers cannot derive valid information from the cipher images. Table 16 lists the $\chi^{2}$ test results, information entropies, and correlation coefficients of the cipher images. It can be seen that the proposed algorithm has good encryption performance for all-white and all-black images. Therefore, the proposed algorithm can effectively resist chosen-plaintext and known-plaintext attacks.

### 7.8. Cropping Attack and Noise Attack Analysis

**Figure 18:**
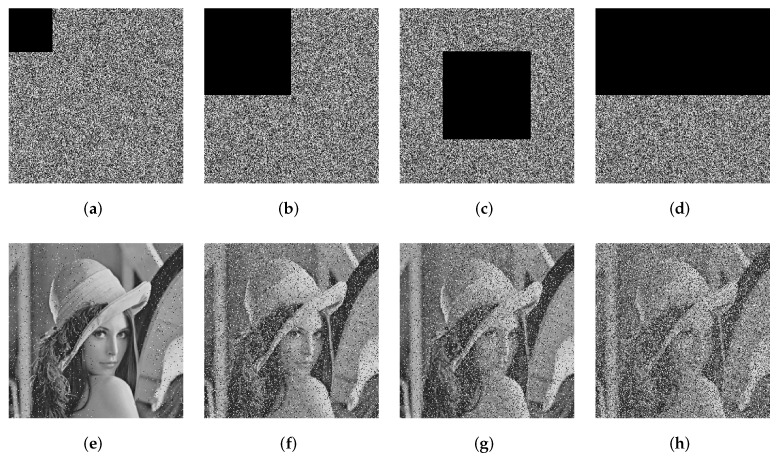
The results of cropping attack. (**a**) Encrypted image with 6.25% cropping, (**b**) encrypted image with 25% cropping, (**c**) encrypted image with 25% cropping (middle), (**d**) encrypted image with 50% cropping, (**e**) decryption of (**a**), (**f**) decryption of (**b**), (**g**) decryption of (**c**), and (**h**) decryption of (**d**).

In the actual transmission process of the network, the images are at high risk of data loss or noise contamination. Therefore, a secure image encryption algorithm shall be robust against cropping attacks and noise. Take “Lena” as a test image. The cropped images are shown in Figure 18a–d. We can see that even if cropping attacks on cipher images lead to data loss, the decrypted image can still be recognized by the human eye. This shows that the proposed algorithm is resistant to cropping attacks.

**Figure 19:**
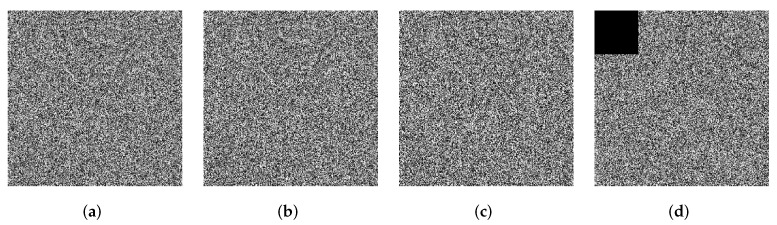

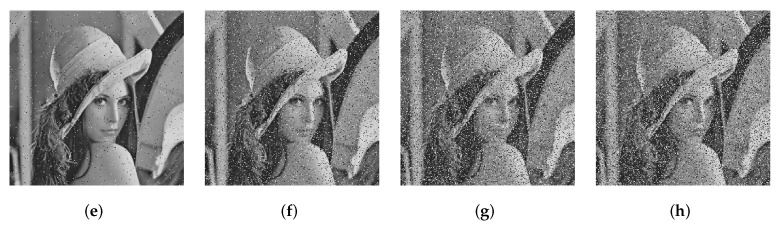
The results of noise attack. (**a**) Encrypted image with 0.01 salt & pepper noise, (**b**) encrypted image with 0.05 salt & pepper noise, (**c**) encrypted image with 0.1 salt & pepper noise, (**d**) encrypted image with 0.05 salt & pepper noise and 6.25% clipping, (**e**) decryption of (**a**), (**f**) decryption of (**b**), (**g**) decryption of (**c**), and (**h**) decryption of (**d**).

To test the antinoise performance of the proposed algorithm, we add salt and pepper noise with different intensities to the cipher image, where the intensities are 0.01, 0.05, and 0.1, respectively. The results are shown in Figure 19a–c. It can be seen that the decrypted images contain some noises, but we can still recognize most of the information in the plain image by human eyes. The proposed algorithm is resistant to noise attacks. In addition, as shown in Figure 19d, salt and pepper noise with intensity of 0.05 is added to the cipher image with 6.25% cropping. The decrypted image Figure 19h can still be recognized by human eyes. Thus, the proposed algorithm can effectively resist cropping attacks and noise attacks.

## 8. Conclusions

In this paper, we develop a hybrid domain image encryption algorithm based on improved Henon map. First, we construct an improved Henon map called 2D-ICHM, and dynamical analysis indicates that it has excellent chaotic properties. Second, an image encryption algorithm with a double sandwich structure is proposed using 2D-ICHM, where the content structure of the image is destroyed by the proposed chunking–arrangement–combination operation, which enhances the security performance of the algorithm. Third, the SHA-512 hash value of the plain image is used to obtain the initial values and system parameters of the chaotic system, which enhances the plaintext sensitivity. Simulation experiments and security analysis show that the proposed image encryption algorithm has a huge key space, strong key sensitivity, and strong robustness to various cryptanalytic attacks. Therefore, the proposed algorithm has high level of security.

However, the limitations of this algorithm include the inability to encrypt color images directly and the unsuitability for real-time confidential communications. We will extend our approach based on the ideas of the block and nature-inspired optimization techniques from the literature [60] to address these shortcomings in future research. Considering the excellent properties of hyperchaotic systems, we try to design a 2D hyperchaotic system for image encryption. In the last few years, machine learning and deep learning networks have shown great advantages in the field of image processing. We attempt to introduce these techniques to simplify and improve the proposed double sandwich encryption structure to design a real-time secure color image encryption algorithm.

## Figures and Tables

**Table 1:** SP800-22 test.

Statistical Test	p_Value
Frequency	0.9856
Block Frequency	0.8178
Cumulative Sums	0.2113
Runs	0.1421
Longest Run	0.6101
Rank	0.3482
Fft	0.5341
Nonoverlapping Template	0.9114
Overlapping Template	0.5341
Approximate Entropy	0.3504
Random Excursions	0.6528
Frequency	0.8562
Random Excursions Variant	0.7236
Serial	0.7399
Linear Complexity	0.0179

**Table 2:** DNA coding rules.

Rules	1	2	3	4	5	6	7	8
00	A	A	C	C	G	G	T	T
01	C	G	A	T	A	T	C	G
10	G	C	T	A	T	A	G	C
11	T	T	G	G	G	G	A	A

**Table 3:** DNA addition operations and XOR operations.

+	A	C	G	T	XOR	A	C	G	T
A	C	A	T	G	A	C	A	T	G
C	A	C	G	T	C	A	C	G	T
G	T	G	A	C	G	T	G	C	A
T	G	T	C	A	T	G	T	A	C

**Table 4:** Key space for different algorithms.

Algorithms	Proposed	Ref. [6]	Ref. [16]	Ref. [51]	Ref. [52]	Ref. [53]	Ref. [54]
Key spaces	$2^{714}$	$2^{99}$	$2^{598}$	$2^{213}$	$2^{186}$	$2^{496}$	$2^{512}$

**Table 5:** Values of NPCR and UACI of Lena’s cipher images.

Secret Keys			Calculated Values	
			NPCR%	UACI%
$v_{1}$	$v_{3}$	*K*	0	0
$v_{1}$	$v_{3}$	$K_{1}$	99.61	33.55
$v_{1}^{\prime }=v_{1}+10^{-14}$	$v_{3}$	*K*	99.60	33.45
$v_{1}$	$v_{3}^{\prime }=v_{3}-10^{-14}$	*K*	99.62	33.46
Ideal value			99.61	33.46

**Table 6:** Values of NPCR and UACI of Lena’s decrypted images.

Secret Keys			Calculated Values	
			NPCR%	UACI%
$v_{1}$	$v_{3}$	*K*	0	0
$v_{1}$	$v_{3}$	$K_{1}$	99.62	28.65
$v_{1}^{\prime }=v_{1}+10^{-14}$	$v_{3}$	*K*	99.65	28.57
$v_{1}$	$v_{3}^{\prime }=v_{3}-10^{-14}$	*K*	99.58	28.60
Ideal value			99.61	28.62

**Table 7:** $\chi^{2}$ test.

Image	Plain	Cipher
Lena	$4.2698\times 10^{4}$	231.1174
Peppers	$1.2892\times 10^{5}$	271.2109
4.1.01	$3.0295\times 10^{5}$	271.6172
4.1.02	$7.1297\times 10^{5}$	258.7578
4.1.03	$1.4396\times 10^{6}$	230.5156
5.1.09	$1.3569\times 10^{5}$	219.5625
5.1.10	$5.0863\times 10^{4}$	240.9844
5.1.11	$2.2085\times 10^{5}$	258.7188
5.1.12	$2.8206\times 10^{5}$	275.6250
5.1.13	$1.1983\times 10^{7}$	239.3359
5.1.14	$5.0326\times 10^{4}$	251.0156
6.1.01	$1.2230\times 10^{5}$	225.1953

**Table 8:** Correlation coefficients of images.

Image	Cipher Image			Plain Image		
	Horizontal	Verticall	Diagonal	Horizontal	Vertical	Diagonal
Lena	−0.0008	0.0041	−0.0011	0.9753	0.9425	0.9180
Peppers	−0.0006	0.0052	−0.0043	0.9848	0.9906	0.9714
4.1.01	0.0010	−0.0016	−0.0123	0.9625	0.9672	0.9462
4.1.02	0.0045	−0.0012	−0.0121	0.9558	0.9312	0.8956
4.1.03	0.0017	0.0014	0.0068	0.9166	0.9729	0.9092
5.1.09	0.0035	−0.0023	−0.0037	0.9397	0.9008	0.9038
5.1.10	0.0042	−0.0052	0.0017	0.9399	0.9640	0.8977
5.1.11	0.0023	0.0021	−0.0020	0.8583	0.9061	0.8207
5.1.12	0.0006	−0.0108	0.0138	0.9750	0.9568	0.9367
5.1.13	−0.0019	0.0008	0.0028	0.8756	0.8750	0.7585
5.1.14	−0.0026	−0.0056	−0.0013	0.8962	0.9454	0.8540
6.1.01	0.0156	−0.0015	0.0071	0.9906	0.9872	0.9751

**Table 9:** Comparison on correlation coefficients for Lena.

Algorithms	Horizontal	Vertical	Diagonal
proposed	−0.0008	0.0041	−0.0011
Ref. [6]	−0.0209	0.0528	−0.0099
Ref. [16]	0.0058	−0.0024	0.0012
Ref. [51]	0.0082	−0.0032	−0.0025
Ref. [52]	0.0083	−0.0021	−0.0025
Ref. [53]	−0.0021	0.0009	0.0003
Ref. [54]	−0.0148	0.0106	0.0134

**Table 10:** Information entropy of images.

Image	Plain	Cipher
Lena	7.4116	7.9975
Peppers	7.7448	7.9970
4.1.01	7.0525	7.9973
4.1.02	6.4207	7.9972
4.1.03	5.5939	7.9975
5.1.09	6.7093	7.9976
5.1.10	7.3118	7.9973
5.1.11	6.4523	7.9971
5.1.12	6.7057	7.9970
5.1.13	1.5483	7.9974
5.1.14	7.3424	7.9972
6.1.01	7.2044	7.9975

**Table 11:** Information entropy comparison of Lena’s cipher image.

Image	Proposed	[6]	[16]	[51]	[52]	[53]	[54]
Lena	7.9975	7.9661	7.9975	7.988	7.9971	7.9971	7.9975

**Table 12:** The critical values of NPCR and UACI.

Images Size	NPCR%			UACI%		
	$N_{0.05}$	$N_{0.01}$	$N_{0.001}$	$\left(U_{0.05}^{-},U_{0.05}^{+}\right)$	$\left(U_{0.01}^{-},U_{0.01}^{+}\right)$	$\left(U_{0.001}^{-},U_{0.001}^{+}\right)$
256×256	99.5693	99.5527	99.5341	(33.2824, 33.6447)	(33.2255, 33.7016)	(33.1594, 7677)

**Table 13:** NPCR test value.

Image	NPCR%	Critical NPCR%		
		$N_{0.05}=99.5693\%$	$N_{0.01}=99.5527\%$	$N_{0.001}=99.5341\%$
Lena	99.6172	✓	✓	✓
Peppers	99.6086	✓	✓	✓
4.1.01	99.5892	✓	✓	✓
4.1.02	99.5793	✓	✓	✓
4.1.03	99.6002	✓	✓	✓
5.1.09	99.5998	✓	✓	✓
5.1.10	99.6052	✓	✓	✓
5.1.11	99.6175	✓	✓	✓
5.1.12	99.6134	✓	✓	✓
5.1.13	99.6100	✓	✓	✓
5.1.14	99.5895	✓	✓	✓
6.1.01	99.6213	✓	✓	✓
All black	99.5987	✓	✓	✓

**Table 14:** UACI test value.

Image	UACI%	Critical UACI%		
		$U_{0.05}^{-}=33.2824\%$	$U_{0.01}^{-}=33.2255\%$	$U_{0.001}^{-}=33.1594\%$
		$U_{0.05}^{+}=33.6447\%$	$U_{0.01}^{+}=33.7016\%$	$U_{0.001}^{+}=33.7677\%$
Lena	33.4516	✓	✓	✓
Peppers	33.4752	✓	✓	✓
4.1.01	33.5487	✓	✓	✓
4.1.02	33.4870	✓	✓	✓
4.1.03	33.3864	✓	✓	✓
5.1.09	33.5019	✓	✓	✓
5.1.10	33.5172	✓	✓	✓
5.1.11	33.2873	✓	✓	✓
5.1.12	33.5091	✓	✓	✓
5.1.13	33.4249	✓	✓	✓
5.1.14	33.5100	✓	✓	✓
6.1.01	33.5516	✓	✓	✓
All black	33.4624	✓	✓	✓

**Table 15:** NPCR and UACI values of Lena for different algorithms.

Algorithms	NPCR%	UACI%
Proposed	99.6172	99.4516
Ref. [6]	-	-
Ref. [16]	99.60	33.45
Ref. [51]	99.6150	33.4205
Ref. [52]	-	-
Ref. [53]	99.9596	33.4588
Ref. [54]	99.5041	33.4238

**Table 16:** Encryption results of all white and black images.

Cipher Image	$\chi^{2}$ Test	Information Entropy	Correlation Coefficients		
			Horizontal	Vertical	Diagonal
All white	272.6641	7.9970	0.0008	−0.0005	0.0087
All black	253.8516	7.9972	−0.0012	−0.0019	0.0116

## Data Availability

Data sharing not applicable.

## Abbreviations

The following abbreviations are used in this manuscript:

IWT	Integer wavelet transform
DNA	Deoxyribonucleic acid
SHA-512	Secure hash algorithm-512
DES	Data Encryption Standard
AES	Advanced Encryption Standard
LSTM-ANN	Long short-term memory artificial neural networks
GAN	Generative adversarial network
CNN	Convolutional neural network
1D	One-dimensional
2D	Two-dimensional
HD	High-dimensional
2D-CHM	Classical two-dimensional Henon map
2D-ICHM	Improved classical two-dimensional Henon map
2D-SM	Two-dimensional sine map
IHM	Improved Henon map
QR	Qatari Rial
ApEn	Approximate entropy
LE	Lyapunov exponent
A	Adenine
C	Cytosine
G	Guanine
T	Thymine
NPCR	Number of pixels change rate
UACI	Unified average changing intensity
